# The impact of maternal incarceration on mothers and children: A scoping review protocol

**DOI:** 10.1371/journal.pone.0355209

**Published:** 2026-08-28

**Authors:** Lynn Fenton, Daragh Bradshaw, Rose Galvin, Luke Slattery, Fiona Donson, Aisling Parkes, Leonie Ludwig, Elaine Rogers, Srijani Bose, Anca Minescu

**Affiliations:** 1 Department of Psychology, Centre of Social Issues Research, University of Limerick, Limerick, Ireland; 2 School of Allied Health, University of Limerick, Limerick, Ireland; 3 Department of Law, University College Cork, Cork, Ireland; University of North Carolina at Chapel Hill, UNITED STATES OF AMERICA

## Abstract

Globally, female incarceration rates have risen in recent decades. Estimates indicate that between 60–80% of incarcerated women are mothers. Maternal incarceration (MI) is associated with negative and often enduring implications for both the mother and child. Recognising the risk of harmful outcomes, evidence-based support for both populations is essential. However, this broad research topic draws interest from academics and practitioners across many distinct but overlapping disciplines, each with different priorities, and methodological and theoretical approaches. Interdisciplinary collaboration remains rare. Consequently, available evidence is spread across different platforms and lacks overview, making it difficult to identify and access key findings, or establish where research gaps exist. Together, these factors hinder researchers and practitioners and constrain efforts to develop evidence‑based support, a task often undertaken by non-governmental organisations already operating with limited resources. Addressing the current lack of overview, this review aims to systematically map the available primary and secondary evidence. Specifically, we seek to explore the overarching research question: ‘What empirical evidence is available that documents the impact of MI on (a) mothers and (b) children?’ Following the Joanna Briggs Institute Manual and Population/Concept/Context framework, a comprehensive search will be conducted across six multidisciplinary databases. Relevant papers will be exported to Covidence and screened by independent reviewers against pre-established inclusion criteria. Included papers will be mapped and relevant information charted using EPPI-Reviewer. Findings will document the scope of the available evidence and identify gaps in our current understanding, creating a helpful resource that can inform future research, advise policy and practice, and guide evidence-based support.

## Introduction

Recent decades have seen an increase in global female prisoner populations [1]. Approximately 60–80% of female prisoners are mothers [2], transforming a once-rare childhood experience into an increasingly common reality for many children worldwide, particularly those from poor or marginalised backgrounds [3]. This warrants concern, as research indicates that exposure to maternal incarceration is associated with various negative and often long-lasting outcomes for both mother [4–10] and child [11–14], which may compound existing vulnerabilities and perpetuate cycles of disadvantage. Adverse implications are documented across social [4,6,7,9–14], emotional/psychological [4–14], behavioural [4,7,11–14], health [4,7,10–14], educational [14], and economic domains [10].

In recognition of the potential harms associated with MI, along with the pre-existing vulnerabilities many of these individuals face, there has been growing advocacy for enhanced and targeted support for both mother and child [3–6,15]. However, effective support initiatives need to be grounded in evidence. Currently, due to the broad nature of the topic, existing evidence is spread across various distinct but overlapping disciplines, such as law [16], psychology [14,17], criminology [18,19], sociology [20], public health, and medicine [21–23]. Each discipline operates with unique priorities, differing platforms and databases, and distinct methodological and theoretical approaches, with interdisciplinary collaboration limited to date [24–26]. Furthermore, despite rising female incarceration rates globally, existing research has chiefly focused on US samples [7,13,16,27–29], limiting generalisability of findings to other non-US contexts. Consequently, the process of locating and retrieving context-relevant key findings, necessary for the development of effective supports, is further complicated, and often falls to non-governmental organisations, many of which already operate with limited resources.

The present protocol outlines the planned conduct of a scoping review which aims to address the current lack of overview by systematically identifying and mapping the available primary and secondary evidence, addressing the research question ‘What empirical literature is available that documents the impact of MI on mothers and children?’. Given the broad nature of the topic and wide dispersion of existing evidence across disparate disciplines and databases, a scoping review was deemed the most appropriate methodological approach [30]. This methodological approach is particularly suited to the aims of this review, which include mapping the current research landscape, offering an overview of the breadth and depth of the literature, and identifying key gaps in knowledge [30]. As such, this scoping review will serve as a valuable precursor to future systematic reviews by highlighting areas where sufficient evidence exists to warrant systematic synthesis [30] and to confirm the relevance of potential research questions for subsequent investigation.

A preliminary literature search (June 2024) identified two relevant studies that address similar research questions [8,10], and a subsequent search (May 2026) yielded four additional studies [31–33]. However, the proposed review outlined in this protocol differs from these previous studies in several respects (see [S1Appendix]). Most notably, we aim to adopt a broader and more inclusive scope, incorporating evidence arising from qualitative, quantitative, and mixed-methods research studies, as well as systematic reviews and meta-analyses.

## Materials and methods

The review will be guided by the methodological framework proposed by Arksey and O’Malley (2005) [34] and the detailed, up-to-date, evidence-based recommendations outlined in Chapter Ten of the JBI Manual for Evidence Synthesis [35]. Using these resources strengthens the reproducibility of the methods. In accordance with JBI guidelines, the Population/Concept/Context (PCC) framework was used to develop the research questions and search strategy [36]. Applying this framework at the protocol stage supports clear objectives and inclusion criteria and ensures that eventual data extraction is relevant to the research questions [36,37]. Any deviations from the methods outlined by JBI will be clearly stated and justified in the final scoping review.

### Research questions

Developed in accordance with the PCC framework, the research questions for the proposed review are as follows:

1. What empirical evidence is available that documents the impact of MI on mothers?
2. What empirical evidence is available that documents the impact of MI on their children?

### Eligibility criteria

The JBI Manual for Evidence Synthesis was employed in conjunction with the PCC framework to specify the inclusion and exclusion criteria for this review [35]. To strengthen the rigor and credibility of the findings, this review will focus exclusively on peer reviewed publications [38]. To identify relevant available evidence, published, peer-reviewed articles that contain original research, including experimental research, case studies, quantitative, qualitative, or mixed-method approaches will be included, as well as systematic reviews and meta-analyses. As advised by Ghezzi-Kopel and Porciello [39], numbered lists of study inclusion and exclusion criteria (and, where relevant, their alignment with the PCC framework) are provided below.

#### Inclusion criteria.

1. Population:
  a) Mothers (pregnant or have children) who are currently incarcerated, and mothers who were previously incarcerated and were pregnant or had children during their incarceration.
  b) Children whose mother is or has been incarcerated during their lifetime.
2. Concept: The impact of MI, across various domains, including psychological, social, educational, behavioural, and health outcomes.
3. Context: All forms of incarceration, including imprisonment, jail, custody, and correctional facilities, across all countries and regions worldwide (provided that the evidence is available in English).
4. Published, peer-reviewed articles that contain original research, meta-analyses, systematic reviews, experimental research, case studies, or other quantitative, qualitative, or mixed-method approaches.
5. Articles must be published in peer-reviewed journals.
6. Due to language limitations within the research team, articles must be published in English.

#### Exclusion criteria.

1. Population: All incarcerated males, and incarcerated females who are not mothers, do not have children, and are not pregnant. This also includes females who are now mothers but, during their past incarceration, were neither pregnant nor had children. Furthermore, studies that investigate the impact of parental incarceration on mothers or children but do not differentiate between the effects of paternal and MI will also be excluded.
2. Concept: Studies that do not investigate the impact of MI on mothers or their children.
3. Context: Contexts that are not related to incarceration.
4. Non-peer-reviewed articles or otherwise non-empirical studies, including book reviews, opinion pieces, and editorial letters.
5. Studies published in languages other than English.

### Information sources

The multidisciplinary databases PubMed, PsycINFO, CINAHL (on the EBSCO platform), Web of Science, Scopus, and the Campbell Collaboration, have been selected based on their relevance to the topic of interest and associated research questions.

### Search strategy

The final scoping search strategy is informed by the primary concepts of *incarceration* and *outcomes* outlined in Bradshaw et al., 2025 [40]. The strategy was subsequently refined to align with the specific research questions of the review and further supplemented by guidance from a librarian specialising in education and health sciences research. It also incorporates insights from a preliminary database search, during which keywords were identified through screening titles and abstracts, examining index terms, and reviewing the reference lists of relevant articles.

Final search terms align with the population *(incarcerated mothers and their children)* and context *(the experience of MI)* categories of the PCC framework. To ensure our search remains comprehensive, the decision was made to exclude any terminology related to the concept category *(impact; across various domains like social, psychological, behavioural, educational and health)*, as it was considered too restrictive and posed a risk of missing relevant research. The following search “(mater* OR mother* OR mum* OR mom* OR pregn*) AND (prison* OR detain* OR detention OR offend* OR correction* OR incarcerat* OR jail* OR imprison* OR gaol*) AND (child* OR adolesce* OR teen* OR youth* OR young* OR minor* OR kid* OR student*)” will be applied to the title, abstract, and keyword fields of each database. A full database search conducted on APA PsycINFO (EBSCO) can be viewed in [S2 Appendix**].**

### Study records

#### Data management.

The software Covidence and EPPI-Reviewer will be employed to manage data throughout the review process and map findings. Articles found using our search strategy will be imported to Covidence for duplication removal, screening, and record keeping. Included papers will be imported to EPPI-Reviewer, where relevant information will be charted and mapped. To ensure the replicability of our search and selection processes, data will be documented utilising the Preferred Reporting Items for Systematic reviews and Meta-Analyses extension for Scoping Reviews checklist (PRISMA-ScR) [41]. A sample of this checklist can be found in [S3Appendix].

#### Selection process.

After the identified articles are imported to Covidence and duplicates removed, titles and abstracts will be screened by two independent reviewers against the inclusion criteria for the review. Potentially relevant papers as approved by both reviewers will be retrieved in full. The full text of these studies will then be examined. Rationale for excluding full text studies will be systematically documented and stated in the final scoping review. Any conflicts which may arise between the reviewers during the selection process will be resolved through discussion and, if required, the input of an independent third reviewer. Search results will be reported both narratively and in a PRISMA flow diagram [41]. A sample of this diagram can be found in [S4Appendix].

#### Data charting.

Microsoft Excel and EPPI-Reviewer will be used for data charting, as extraction tools within Covidence are best suited for reviews of clinical trials using the PICO framework. An initial data charting tool (see supplementary material) has been developed, in line with the *population, context,* and *concept* (PCC) framework, as per best practice guidelines outlined in Chapter Ten of JBI Manual for Evidence Synthesis [35] and tailored for relevancy to the review questions. While primary data items of interest are structured around *population, context,* and *concept*, additional data describing *study characteristics* will also be extracted, as these data are directly relevant to the aims of this review and will support mapping of the current research landscape [37]. Specific data items include (a) *population*; whether the primary focus was the mother or the child; (b) *context*; (i) country and (ii) stage of incarceration (during incarceration, or post-incarceration); (c) *concept*; key impacts or outcomes reported; and (d) *study characteristics;* including (i) title, (ii) author (iii) year of publication, (iv) field of publication, (v) methodological approach (qualitative, quantitative, mixed methods or systematic review) and (vi) reported theoretical underpinnings. These criteria may be modified with the addition of previously unidentified themes or points of interest should they emerge during the piloting phase. Any modifications will be documented in the final scoping review.

Piloting will be carried out over an 8-week period, whereby the charting tool will be trialled on a proportion of the included articles (15%). Piloting will be conducted by members of the research team, who will meet weekly with the overall research team, to discuss and resolve emerging queries. The tool will be iteratively refined through these weekly discussions to ensure all key information from the articles is being captured. Once satisfied with the scope of the extraction tool, inter-rater reliability checks will be conducted by an independent reviewer on a subset of the included articles (10%). Once acceptable inter-rater reliability scores are achieved (>0.61) [42], the remaining data extraction will be carried out. Weekly team meetings will remain constant throughout the data extraction process, where concerns can be raised and resolved through discussion.

### Data synthesis

The results of our search will be consolidated in a PRISMA flow diagram [41], supported by a concise narrative description, and accompanying visual representation. In accordance with JBI guidance [35] and other published scoping reviews [43–47], secondary evidence such as systematic reviews, will be included to map existing secondary evidence and to characterize how the topic has previously been synthesized. Potentially relevant evidence of this kind will be documented transparently, with data extraction confined to descriptive information regarding their scope, methods, and impact focus [35]. Primary findings from articles captured within these systematic reviews will not be re-extracted, thereby avoiding duplication of evidence. Both primary and secondary evidence will be labelled accordingly, ensuring clarity and transparency.

## Discussion

This scoping review protocol outlines a proposed systematic approach to identifying and mapping peer-reviewed, published literature on the impact of MI on mothers and children. The review will provide researchers, stakeholders, and policymakers with a comprehensive overview of current evidence, signposting key themes, as well as gaps in existing literature. Through signposting and mapping available evidence, areas with substantial primary research will emerge and can inform subsequent systematic syntheses, while simultaneously drawing attention to evidence gaps that merit further investigation. Mapping the current evidence base can also help reduce unnecessary duplication of research efforts and minimise resource waste. This is particularly important for secondary evidence, as by highlighting areas that have already been synthesised, this review will enable researchers to allocate resources to topics that remain un-synthesised, thereby supporting more strategic research planning. Furthermore, the review will also have implications for policy and practice, supporting evidence-informed decision-making, intervention design, and implementation.

The planned methodology is designed to ensure transparency and reproducibility. However, it is possible that relevant sources may be missed due to language restrictions. The review will only include articles available in English, meaning relevant articles published in other languages will be excluded. Moreover, to enhance rigour and credibility of findings, the review is limited to published peer-reviewed articles. While this strengthens the quality of included evidence, potentially relevant findings that are not in the form of peer-reviewed publication may be overlooked.

In conclusion, by systematically identifying and mapping available evidence and highlighting critical gaps, this review will provide a comprehensive overview of the available knowledge on the impact of MI on mothers and children. The findings of this review will have important implications for future research, policy and practice, supporting evidence‑based decision‑making and, ultimately, improving support for mothers and children affected by MI.

## Supporting information

S1 Appendix Comparison of the current review with existing reviews. (DOCX)

S2 Appendix Full database search on APA PsycINFO (May 2026). (DOCX)

S3 Appendix PRISMA-P-checklist. (PDF)

S4 Appendix PRISMA flow diagram. (DOCX)

S5 Appendix Data extraction tool. (XLSX)
