# Supplementary material for: The impact of maternal incarceration on mothers and children: A scoping review protocol
Source: PLoS One. 2026 Aug 28;21(8):e0355209. doi: 10.1371/journal.pone.0355209 (PMC13524290; doi:10.1371/journal.pone.0355209)
Supplement: Supplementary file 2 [file pone.0355209.s001.docx]

**Appendix A**

*Comparison of the Current Review with Previous Reviews*

|  | **Title of Publication** | **Author(s)** | **Key Differences** |
| --- | --- | --- | --- |
| 1. | *Experiences of mothering from prison; a qualitative evidence synthesis* | Walsh et al. | The proposed review will incorporate mixed methods, rather than solely qualitative, enabling a more comprehensive understanding of the topic |
| 2. | *The Lived Experience of Motherhood after Prison: A Qualitative Systematic Review* | Gobena et al. | The proposed review will incorporate mixed methods, rather than solely qualitative, as well as research documenting the experience of maternal incarceration during incarceration in addition to the post release experience. |
| 3 | *Mental Disorders Among Mothers in Contact with the Criminal Justice System: A Scoping Review and Meta-analysis* | Sapkota et al. | The proposed review will include impact and outcomes across a variety of domain beyond diagnosed mental disorders. |
| 4. | *How do children describe their experiences of parental imprisonment: A qualitative scoping review* | Zhang & Flynn | The proposed review will incorporate both qualitative and quantitative findings, offering more extensive insights into the impact of MI on children. |
| 5. | *The impact of maternal versus paternal imprisonment on their children’s health: A scoping review* | Gadian et al. | The proposed review will consist of studies which explore the impact of MI without the requirement of having a comparison group included. |
| 6. | *The Birth Experience of Women in the Czech Prison System* | Lochmannová et al. | The proposed review will incorporate this experience of all mothers, not just birthing mothers, and in countries beyond Czechia. |
