# Supplementary material for: The impact of maternal incarceration on mothers and children: A scoping review protocol
Source: PLoS One. 2026 Aug 28;21(8):e0355209. doi: 10.1371/journal.pone.0355209 (PMC13524290; doi:10.1371/journal.pone.0355209)
Supplement: Supplementary file 3 [file pone.0355209.s002.docx]

**Appendix B**

*Full database search: APA PsycInfo (May 2026)*

| **Step** | **Search Fields** | **Search Syntax** | **Total Number Articles Identified** |
| --- | --- | --- | --- |
| 1. | Title | (mater* OR mother* OR mum* OR mom* OR pregn*) | 97,623 |
| 2. | Abstract | (mater* OR mother* OR mum* OR mom* OR pregn*) | 415,859 |
| 3. | Keywords | (mater* OR mother* OR mum* OR mom* OR pregn*) | 121,333 |
| 4. |  | 1 OR 2 OR 3 | 434,950 |
| 5. | Title | (prison* OR detain* OR detention OR offend* OR correction* OR incarcerat* OR jail* OR imprison* OR gaol*) | 52,509 |
| 6. | Abstract | (prison* OR detain* OR detention OR offend* OR correction* OR incarcerat* OR jail* OR imprison* OR gaol*) | 130,563 |
| 7. | Keywords | (prison* OR detain* OR detention OR offend* OR correction* OR incarcerat* OR jail* OR imprison* OR gaol*) | 52,538 |
| 8. |  | 5 OR 6 OR 7 | 146,690 |
| 9. | Title | (child* OR adolesce* OR teen* OR youth* OR young* OR minor* OR kid* OR student*) | 875,934 |
| 10. | Abstract | (child* OR adolesce* OR teen* OR youth* OR young* OR minor* OR kid* OR student*) | 1,743,122 |
| 11. | Keywords | (child* OR adolesce* OR teen* OR youth* OR young* OR minor* OR kid* OR student*) | 942,572 |
| 12. |  | 9 OR 10 OR 11 | 1,926,450 |
| 13. |  | 4 AND 8 AND 12 | 5,284 |
